# Polygodial and Ophiobolin A Analogues for Covalent Crosslinking of Anticancer Targets

**DOI:** 10.3390/ijms222011256

**Published:** 2021-10-19

**Authors:** Vladimir Maslivetc, Breana Laguera, Sunena Chandra, Ramesh Dasari, Wesley J. Olivier, Jason A. Smith, Alex C. Bissember, Marco Masi, Antonio Evidente, Veronique Mathieu, Alexander Kornienko

**Affiliations:** 1 Department of Chemistry and Biochemistry, Texas State University, San Marcos, TX 78666, USA; breana.laguera@emory.edu (B.L.); sunena2293@yahoo.com (S.C.); rameshdiict2@gmail.com (R.D.); 2 School of Natural Sciences-Chemistry, University of Tasmania, Hobart, TAS 7001, Australia; wesley.olivier@utas.edu.au (W.J.O.); jason.smith@utas.edu.au (J.A.S.); alex.bissember@utas.edu.au (A.C.B.); 3 Dipartimento di Scienze Chimiche, Università di Napoli Federico II, Complesso Universitario Monte Sant’Angelo, Via Cintia 4, 80126 Napoli, Italy; marco.masi@unina.it (M.M.); evidente@unina.it (A.E.); 4 Department of Pharmacotherapy and Pharmaceutics, Faculté de Pharmacie, Université Libre de Bruxelles (ULB), 1050 Brussels, Belgium; Veronique.Mathieu@ulb.be; 5 UCRC, ULB Cancer Research Center, Université Libre de Bruxelles (ULB), 1050 Brussels, Belgium

**Keywords:** anticancer activity, apoptosis resistance, ophiobolin A, polygodial, Wittig reaction

## Abstract

In a search of small molecules active against apoptosis-resistant cancer cells, including glioma, melanoma, and non-small cell lung cancer, we previously prepared α,β- and γ,δ-unsaturated ester analogues of polygodial and ophiobolin A, compounds capable of pyrrolylation of primary amines and demonstrating double-digit micromolar antiproliferative potencies in cancer cells. In the current work, we synthesized dimeric and trimeric variants of such compounds in an effort to discover compounds that could crosslink biological primary amine containing targets. We showed that such compounds retain the pyrrolylation ability and possess enhanced single-digit micromolar potencies toward apoptosis-resistant cancer cells. Target identification studies of these interesting compounds are underway.

## 1. Introduction

Cancers with intrinsic resistance to apoptosis are characterized by the lack of responsiveness to the current chemotherapeutic agents that generally work by the induction of apoptosis in cancer cells. These cancers include tumors of the lung, melanoma and glioblastoma and they represent a major challenge in the clinic [1]. For example, patients afflicted by glioblastoma multiforme [2,3], have a median survival expectancy of less than 14 months when treated with the best available protocol that involves surgery, radiation and chemotherapy with temozolomide [4]. Further, the main cause of death of cancer patients are tumor metastases. Metastatic cells are resistant to anoikis, a type of apoptotic cell death triggered by the loss of contact with the extracellular matrix or neighboring cells [5]. Resistance to anoikis, and thus apoptosis, renders metastatic cells unresponsive to the large majority of proapoptotic agents as well [3,6]. Therefore, a search for novel anticancer agents that can overcome cancer cell resistance, including resistance to apoptosis, is an important pursuit.

In this connection, natural products represent a valuable source of not only cytotoxic compounds, but also those capable of overcoming the intrinsic resistance of cancer cells to apoptosis [7,8,9,10]. Half of the anticancer drugs developed since the 1940s either originated from natural sources, such as vinca alkaloids, taxanes, podophyllotoxin, and camptothecins; or synthetic derivatization of natural products, such as vinflunine (fluorinated vinca alkaloid derivative), cabazitaxel (derivative of a natural taxoid), and mifamurtide (derivative of muramyl dipeptide) [11,12].

In addition to their ability to infiltrate a variety of cell processes and locales, natural products are known to be able to modulate multiple molecular targets that are frequently deregulated in cancers. This can be especially useful for overcoming the resistance of cancer cells to single-target pharmaceutical drugs [13,14]. While apoptosis is the most widespread and well-studied mode of cell death induced by synthetic and naturally occurring anticancer agents, alternative mechanisms of cell death (including, autophagy, regulated necrosis, mitotic catastrophe, paraptosis, parthanatos, methuosis, and lysosomal membrane permeabilization) are prevalent among natural products [15,16,17].

**Figure 1:**
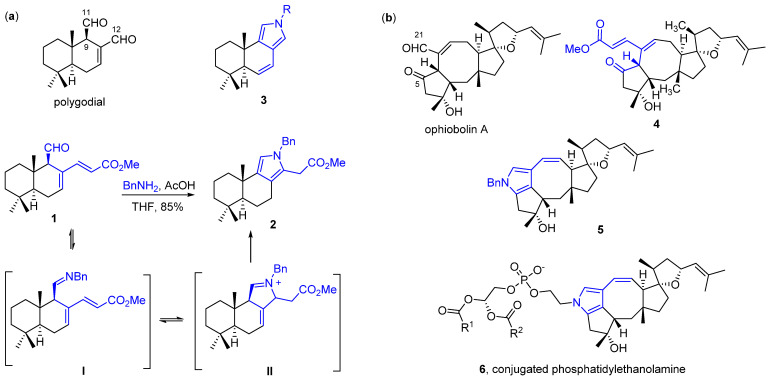
(**a**) Structures of polygodial, Wittig product **1**, a proposed mechanism for pyrrole **2** formation involving intermediates I and II, unstable pyrrole **3**; (**b**) Structures of ophiobolin A, Wittig product **4,** pyrrole **5** and conjugated phosphatidylethanolamine **6**.

In the pursuit of agents active against apoptosis- and multidrug-resistant cancer cells, our recent studies have focused on the synthetic derivatives of polygodial (Figure 1a), a sesquiterpenoid dialdehyde isolated from *Tasmannia lanceolata*, and ophiobolin A (Figure 1b), a plant toxin isolated from pathogenic fungi of the *Bipolaris genus* (*Drechslera gigantea* and *Bipolaris maydis*). For instance, we have shown that C12-Wittig derivatives of polygodial (**1**) exhibit antiproliferative activity mainly through cytostatic effects and in many cases display higher efficiency compared to the parent compound [18]. Additionally, both polygodial and compound **1** can readily undergo the pyrrole formation with primary amines to give **3** and **2**, which can lead to pyrrolation of lysine residues in biological environment [18,19,20].

Another natural anticancer product, ophiobolin A (Figure 1b), represents an interesting candidate for the treatment of apoptosis-resistant cancer cells due to a unique mechanism of action [21]. It was demonstrated to induce paraptosis in glioblastoma cells, offering an innovative strategy to combat this aggressive cancer. This cellular effect is understood to also originate from pyrrolylation of primary amine groups abundant in human cells (**5**) [22], such as intracellular lysine residues or phosphatidylethanolamine (**6**), which would lead to lipid bilayer destabilization [23]. Similarly to polygodial derivative **1**, ophiobolin A can be converted into α,β-unsaturated ester **4** when treated with stabilized Wittig reagents [22].

**Figure 2:**
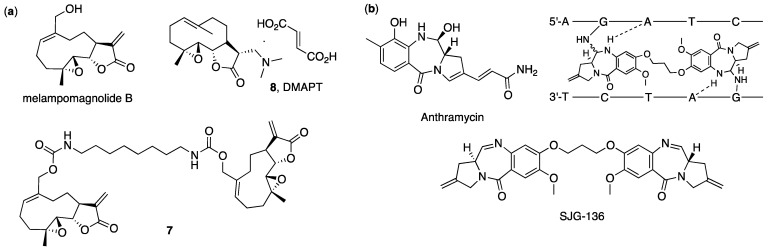
Dimeric anticancer conjugates of natural products: (**a**) Melampomagnolide B, its monomeric (**8**) and dimeric (**7**) derivatives; (**b**) Anthramycin, dimer SJG-136 and schematic representation of interstrand cross-link formation through DNA binding.

The work reported in the current manuscript describes the early attempts of improving anticancer activity of polygodial and ophiobolin A derivatives through preparation of synthetic analogues that are capable of the covalent crosslinking of anticancer targets via the pyrrolylation of primary amines. The method that we envisioned involves the construction of dimeric conjugates of above-mentioned natural products. This approach has a proven record of success in oncology: such dimeric conjugates were often found to possess significantly improved anticancer activity compared to parent compounds (Figure 2) [24,25,26,27,28,29,30,31].

For instance, Crooks and coworkers [24] synthesized dimers of anticancer sesquiterpene lactone melampomagnolide B (Figure 2a) and assessed their activity against the NCI panel of 60 human hematological and solid tumor cell lines. Most active products exhibited potent growth inhibition (GI_50_ = 0.16–0.99 μM) against the majority of cells. Notably, some of the compounds (**7**) displayed up to 1 × 10^6^-fold higher cytotoxic effect against rat 9L-SF gliosarcoma cells when compared to previously developed monomeric melampomagnolide B analogs, such as DMAPT (**8**). This remarkable increase in activity for dimeric structures was attributed to the ability to provide multiple covalent interaction opportunities with multiple exposed surface cysteine residues of the GCLC and GCLM proteins via the formation of Michael addition adducts.

Another well-known example of rationally designed anticancer dimers involves the synthetic modification of the naturally occurring pyrrolobenzodiazepine (PBD) antitumor antibiotics, such as Anthramycin (Figure 2b) [25,26]. These compounds are sequence-selective DNA minor-groove binding agents. When monomeric PBD units were linked to form dimers containing two alkylating imine functionalities, it allowed them to form interstrand or intrastrand DNA cross-links in addition to mono-alkylated adducts, thus resulting in significantly greater cytotoxicity, antitumor activity and antibacterial activity compared to PBD monomers due to the different mode of DNA damage (Figure 2b) [25]. One of these dimers, SJG-136, has displayed potent cytotoxicity in the low nM range against human tumor cell lines and reached Phase II clinical trials in ovarian cancer and leukemia. Subsequent structural modification of the PBD dimers resulted in compounds with enhanced potency through increased interstrand DNA cross-linking ability, including agents with pM and in some cases sub-pM, activity in vitro [26]. These compounds were also employed as chemical payloads of antibody–drug conjugates, some of which are now in Phase III clinical trials [31].

## 2. Results and Discussion

**Figure 3:**
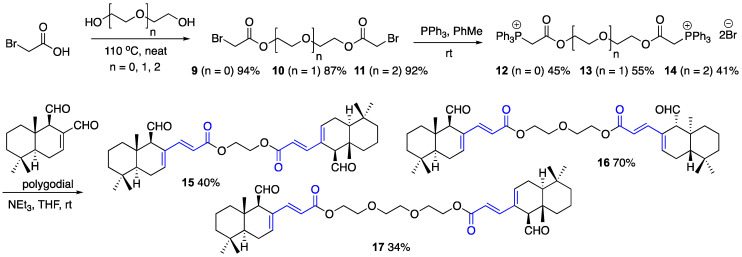
Synthesis of dimeric polygodial-based crosslinking agents **15**, **16** and **17**.

In order to reduce the concept of multivalent anticancer conjugates to practice, in this work we focused on the preparation and assessment of dimeric polygodial and ophiobolin A α,β-unsaturated esters [32,33]. The synthesis of polygodial esters was initiated by the preparation of bromoacetates **9**, **10** and **11** with varied ethylene glycol chain lengths by heating diethylene, triethylene and tetraethylene glycols with bromoacetic acid under neat conditions (Figure 3). Bromoacetates **9**, **10** and **11** were then reacted with 2 eq of triphenylphosphine in toluene at room temperature to give phosphonium bromides **12**, **13** and **14**, respectively, which were conveniently purified with column chromatography on silica gel. Finally, the latter were subjected to Wittig olefination with polygodial, to yield dimeric α,β- and γ,δ-unsaturated esters **15**, **16** and **17** with varied linker chain lengths based on the number of incorporated ethylene glycol moieties. In a manner similar to the preparation of monomeric α,β- and γ,δ-unsaturated esters, such as **1** [18], polygodial reacted at the C-12 aldehyde only, possibly due to the sterically congested nature of the C-11 aldehyde group.

**Figure 4:**
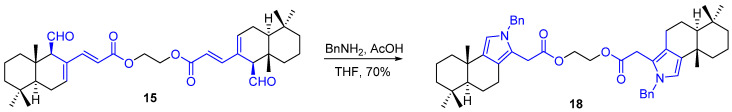
Demonstration of the feasibility of the formation of bis-pyrrole **18** from dimer **15**.

**Figure 5:**
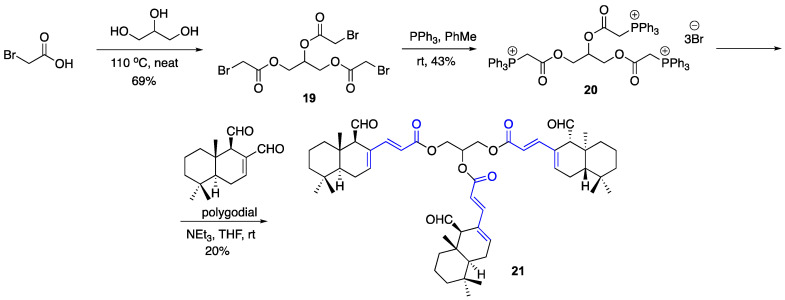
Synthesis of trimeric polygodial-based crosslinking agent **21**.

To confirm that the dimeric unsaturated esters retain the pyrrole forming ability upon the reaction with primary amines such as lysine residues in proteins, compound **15** was reacted with benzyl amine in the presence of AcOH in THF at room temperature and bis-pyrrole **18** was isolated in 70% yield (Figure 4). We also explored the preparation of a possible trimeric unsaturated ester. To this end, glycerol was reacted with bromoacetic acid under neat conditions to give tribromoacetate **19**. The latter was then subjected to a reaction with 3 eq of triphenylphosphine and phosphonium bromide **20** was isolated and purified with column chromatography on silica gel. The latter was treated with 3 eq of polygodial in the presence of Et_3_N in THF at room temperature to give the desired trimeric α,β- and γ,δ-unsaturated ester **21** (Figure 5).

**Figure 6:**
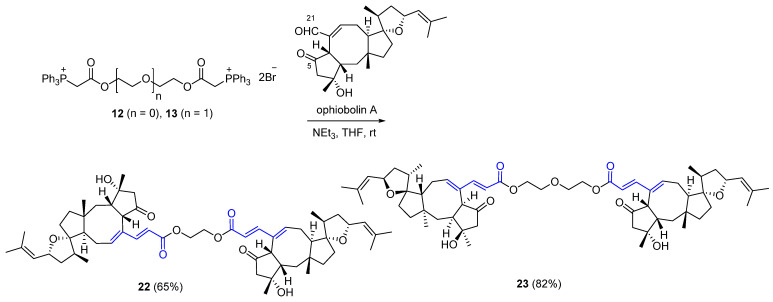
Synthesis of ophiobolin A-based crosslinking agents **22** and **23**.

Ophiobolin A α,β- and γ,δ-unsaturated ester dimers **22** and **23**, containing one and two ethylene glycol unit linkers, respectively, were synthesized using bis(triphenylphosphonium) bromides **12** and **13** (Figure 6). The latter were stirred with excess of ophiobolin A in the presence of Et_3_N in THF at room temperature for 48 h and yielded the desired dimers in good yields.

The synthesized polygodial and ophiobolin A dimeric and trimeric unsaturated esters were evaluated for in vitro growth inhibition using the MTT colorimetric assay against a panel of six cancer cell lines. This included cells resistant to a number pro-apoptotic stimuli, such as human A549 non-small cell lung cancer (NSCLC) [34], human glioblastoma U373 [35,36], and human SKMEL-28 melanoma [37], as well as tumor models, which are largely susceptible to apoptosis-inducing stimuli, such as human Hs683 anaplastic oligodendroglioma [36], human MCF-7 breast adenocarcinoma [38] and mouse B16F10 melanoma (Table 1) [37]. Analysis of these data reveals that where monomeric α,β- and γ,δ-unsaturated esters of polygodial (**1**) and ophiobolin A (**4**) possess double digit micromolar antiproliferative potencies, dimeric polygodial unsaturated ester with one ethylene glycol linker **15** and both ophiobolin unsaturated esters **22** and **23** have single digit micromolar potencies. Thus, the conversion of monomeric covalently reacting polygodial and ophiobolin A unsaturated esters into their dimeric analogues is capable of enhancing the potency by an order of magnitude. Such potencies compare favorably with the clinically used cancer drug cisplatin (Table 1). It is noteworthy that the lengthening of the ethylene glycol linker between the polygodial molecules to two and to three units, i.e., **15**→**16**→**17**, progressively leads to a decrease in the potency of the dimers, pointing to a possible specificity of the interaction between these dimeric agents and their intracellular targets. This specificity is likely to be lost with the trimeric agent **21**, where the potency is in double digit micromolar region as well. Our data are similar to what has been seen in investigations of linker lengths in dimeric agents targeting G protein-coupled receptors [39], estrogen receptors [40], proteolysis targeting chimeras [39], among others [41]. 

**Figure 7:**
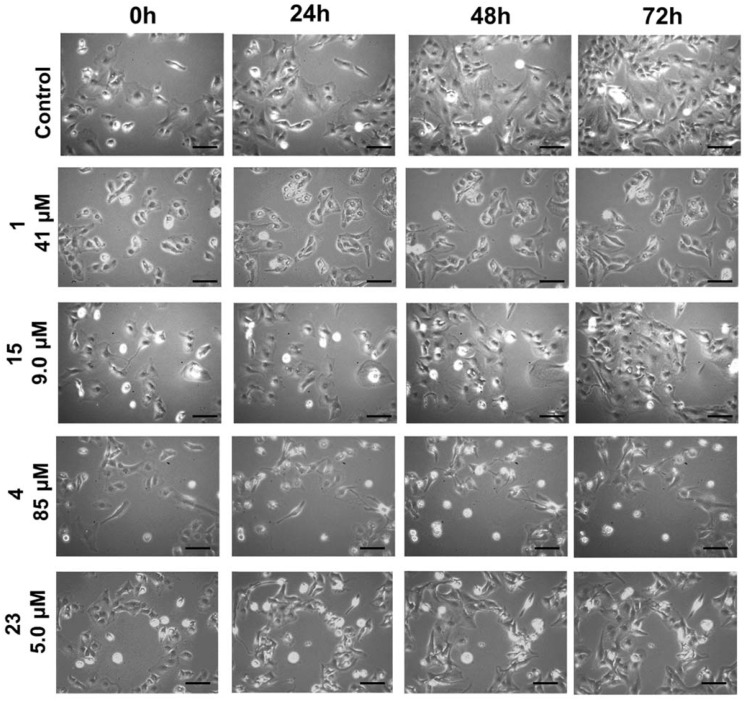
In vitro videomicroscopic analysis of the anticancer effects of monomeric Wittig products **1** and **4** as well as cross-linking agents **15** and **23**. The U373 human glioma cell line was treated with polygodial and ophiobolin A derivatives at their mean GI_50_ concentrations (Table 1) or left untreated. The experiment was conducted once in triplicate. Scale bars correspond to 100 μm.

It is noteworthy that polygodial and ophiobolin A unsaturated esters displayed comparable potencies in cells both sensitive and resistant to apoptosis induction, indicating that this family of compounds is capable of overcoming apoptosis resistance in the clinic (Table 1) [18,22]. The comparison of the cellular effects of monomeric (**1** and **4**) and dimeric (**15** and **23**) polygodial and ophiobolin A esters on human glioblastoma U373 cells by phase contrast videomicroscopy did not reveal marked changes between the monomers and their respective dimers at their respective GI_50_ concentrations (Figure 7). Phenotypically, it thus appears that the mode of action has not changed with the dimerization, despite the increase in potency.

## 3. Materials and Methods

### 3.1. Human Cell Lines and Antiproliferative Effects

Breast carcinoma MCF-7 (DSMZ ACC107), oligodendroglioma Hs683 (ATCC HTB138), non-small cell lung cancer A549 (DSMZ ACC107), glioblastoma U373 (ECACC 08061901), melanoma SKMEL-28 (ATCC HTB72) and the murine melanoma B16F10 (ATCC CRL-6475) were cultured in RPMI supplemented with 10% heat-inactivated FBS (GIBCO code 10270106), 4 mM glutamine (Lonza code BE17-605E), 100 µg/mL gentamicin (Lonza code 17-5182), and penicillin-streptomycin (200 units/mL and 200 µg/mL) (Lonza code 17-602E). Cell lines were cultured in flasks, maintained and grown at 37 °C, 95% humidity, 5% CO_2_. Antiproliferative effects of the compounds on these cell lines were evaluated through the colorimetric assay MTT. Briefly, cells were trypsinized and seeded in 96-well plates. Prior to treatment, compounds were dissolved in DMSO at a concentration of 10 mM. After 24 h, cells were treated with the compounds at different concentrations ranging from 10 nM to 100 µM or left untreated for 72 h. Cell viability was estimated by means of the MTT (3-(4,5-dimethylthiazol-2-yl)-2,5-diphenyl tetrazolium bromide, Sigma, Bornem, Belgium) mitochondrial reduction into formazan in living cells. The optical density of the untreated control was normalized as 100% of viable cells, allowing determination of the concentration that reduced their global growth by 50%.

### 3.2. Natural Product Isolation

Polygodial was isolated from *Tasmannia lanceolata* following a reported procedure [32].

Ophiobolin A was isolated from *Drechslera gigantea* following a reported procedure [33].

### 3.3. Selected Procedure for the Preparation of Polygodial-Based Cross-Linking Agent ***16***

To a solution of Wittig salt **13** (17.5 mg, 0.02 mmol) in THF (2 mL) was added triethylamine (18 μL, 0.13 mmol). The reaction was stirred for 20 min until the Wittig salt dissolved. A solution of polygodial (32.9 mg, 0.14 mmol) was dissolved in THF (0.5 mL) separately and added dropwise to the reaction mixture. The reaction mixture was stirred for 48 h and monitored by TLC (20% ethyl acetate/hexane). The reaction mixture was concentrated and purified by preparative TLC to yield 8.8 mg of **16** (70%). ^1^H NMR (400 MHz, CDCl_3_) δ 9.48 (d, *J* = 4.7 Hz, 2H), 7.33 (d, *J* = 10.6 Hz, 2H), 6.52 (dd, *J* = 8.6, 5.3 Hz, 2H), 5.53 (d, *J* = 16.3 Hz, 2H), 4.36–4.17 (m, 4H), 3.73–3.67 (m, 4H), 2.91–0.81 (m, 38H). ^13^C NMR (500 MHz, CDCl_3_) δ 205.6 (2C), 167.4 (2C), 147.4 (2C), 142.2 (2C), 130.9 (2C), 116.6 (2C), 69.6 (2C), 64.0 (2C), 63.2 (2C), 49.1 (2C), 42.2 (2C), 40.7 (2C), 37.9 (2C), 33.7 (2C), 33.6 (2C), 25.4 (2C), 22.7 (2C), 18.5 (2C), 16.0 (2C). HRMS m/z (ESI) calcd for C_38_H_54_O_7_Na (M+Na) 645.3767, found 645.3769.

### 3.4. Selected Procedure for the Preparation of Ophiobolin A-Based Cross-Linking Agent ***23***

To a solution of Wittig salt **13** (3.1 mg, 3.6 μmol) in THF (2 mL) was added triethylamine (3.2 μL, 0.023 mmol). The reaction was stirred for 20 min until the Wittig salt dissolved. A solution of OpA (10 mg, 0.025 mmol) was dissolved in THF (1 mL) separately and added dropwise to the reaction mixture. The reaction mixture was stirred for 48 h and monitored by TLC (20% ethyl acetate/hexane). The reaction mixture was concentrated and purified by preparative TLC to yield 2.8 mg of **23** (82%). ^1^H NMR (400 MHz, MeOD) δ 7.32 (dd, *J* = 15.6, 0.7 Hz, 2H), 6.43 (t, *J* = 8.5 Hz, 2H), 5.97 (d, *J* = 15.6 Hz, 2H), 5.23–5.16 (m, 2H), 4.55–4.45 (m, 2H), 4.27–4.18 (m, 4H), 3.75–3.69 (m, 4H), 3.67–3.62 (m, 2H), 3.60–3.55 (m, 2H), 2.58–2.45 (m, 4H), 2.45–2.36 (m, 2H), 2.25–2.21 (m, 2H), 2.19–2.06 (m, 6H), 1.87–1.77 (m, 2H), 1.74 (d, *J* = 1.2 Hz, 6H), 1.70 (d, *J* = 1.2 Hz, 6H), 1.67–1.63 (m, 2H), 1.62–1.39 (m, 8H), 1.37 (s, 6H), 1.33–1.26 (m, 2H), 1.09 (d, *J* = 7.2 Hz, 6H), 0.97–0.89 (m, 2H), 0.88 (s, 6H). ^13^C NMR (100 MHz, MeOD) δ 167.3 (2C), 148.1 (2C), 137.1 (2C), 135.4 (2C), 133.6 (2C), 125.5 (2C), 115.9 (2C), 95.3 (2C), 77.1 (2C), 71.1 (2C), 68.9 (2C), 63.3 (2C), 61.3 (2C), 59.9 (2C), 54.5 (2C), 51.6 (2C), 50.3 (2C), 42.7 (2C), 42.6 (2C), 40.7 (2C), 36.5 (2C), 34.8 (2C), 30.1 (2C), 24.6 (2C), 24.1 (2C), 22.1 (2C), 17.0 (2C), 16.9 (2C), 16.8 (2C). HRMS m/z (ESI) calcd for C_58_H_82_O_11_Na (M+Na) 977.5749, found 977.5164.

## 4. Conclusions

In conclusion, previously we synthesized α,β- and γ,δ-unsaturated esters of polygodial and ophiobolin A with potential covalent reactivity in cancer cells, i.e., pyrrolylation of lysine residues in proteins. In the present work we converted such covalently reacting compounds into dimeric and even trimeric species, capable of crosslinking proteins through such pyrrolylation reactions. Indeed, we showed that such dimeric compounds retain the ability to form pyrroles with primary amines. One selected polygodial dimer with the shortest linker length and both synthesized ophiobolin A dimers possess significantly enhanced antiproliferative potencies compared with their monomeric analogues, supporting this research premise. Mechanistic studies with a possible identification of intracellular targets for these molecules are underway.

## Figures and Tables

**Table 1:** In vitro growth inhibitory effects of cross-linking agents in comparison with their monomeric counterparts.

Compound	GI_50_ In Vitro Values (µM) ^1^					
	Resistant to Apoptosis			Sensitive to Apoptosis		
	A549	SKMEL-28	U373	MCF7	Hs683	B16F10
**1**	30	42	41	36	29	7
**15**	7	14	9	4	4	5
**16**	17	22	28	9	8	9
**17**	55	29	79	36	50	24
**21**	25	24	28	23	17	6
**4** ^2^	59	86	86	43	59	ND
**22**	5	8	32	8	10	4
**23**	4	3	5	3	3	3
cisplatin	7	26	ND	35	7	8

^1^ Mean concentration required to reduce the viability of cells by 50% after a 72 h treatment relative to a control, each experiment performed in sextuplicates, as determined by MTT assay. ^2^ Data are from ref. [22]. ND = not determined.

## Data Availability

Not applicable.
